# Oral phase dysphagia in facial onset sensory and motor neuronopathy

**DOI:** 10.1002/brb3.999

**Published:** 2018-05-21

**Authors:** Mitsuru Watanabe, Wataru Shiraishi, Ryo Yamasaki, Noriko Isobe, Motohiro Sawatsubashi, Ryuji Yasumatsu, Takashi Nakagawa, Jun‐ichi Kira

**Affiliations:** ^1^ Department of Neurology Neurological Institute Graduate School of Medical Sciences Kyushu University Fukuoka Japan; ^2^ Department of Neurological Therapeutics Neurological Institute Graduate School of Medical Sciences Kyushu University Fukuoka Japan; ^3^ Department of Otorhinolaryngology Graduate School of Medical Sciences Kyushu University Fukuoka Japan

**Keywords:** dysphagia, facial onset sensory and motor neuronopathy, neurodegeneration, neuroinflammation, oral phase, prognostic factor

## Abstract

**Introduction:**

Facial onset motor and sensory neuronopathy (FOSMN) is a rare disease whose cardinal features are initial asymmetrical facial sensory deficits followed by bulbar symptoms and spreading of sensory and motor deficits from face to scalp, neck, upper trunk, and upper extremities in a rostral–caudal direction. Although bulbar involvement is frequently observed in FOSMN, dysphagia in these patients has not been fully described. In this study, we aimed to characterize dysphagia as a prognostic factor in FOSMN by investigating our institutional case series.

**Methods:**

We retrospectively reviewed the medical records, including swallowing function tests, of six patients with FOSMN (three men and three women) who were thoroughly examined at Kyushu University Hospital between 1 January 2005 and 30 November 2017.

**Results:**

Average age at onset was 58.5 years; average disease duration was 5.7 years. All patients developed bulbar dysfunction and dysphagia (at an average of 1.8 and 2.6 years from onset, respectively), resulting in choking episodes in three patients, percutaneous endoscopic gastrostomy placement in three, and recurrent aspiration pneumonia in one. Four of five patients evaluated with videofluoroscopic swallowing studies had poor oral retention, leading to bolus flowing into the pharynx before swallowing; the fifth patient showed poor lingual transfer. Fiberoptic endoscopic evaluation of swallowing revealed leakage of blue‐dyed water from the mouth to the pharynx in three patients because of poor oral retention, but only mild pharyngeal phase dysphagia in all four cases evaluated.

**Conclusions:**

Oral phase dysphagia predominates in the early stage of FOSMN.

## 1 INTRODUCTION

Facial onset motor and sensory neuronopathy (FOSMN) was first described by Vucic et al. in 2006 (Vucic et al., 2006). Its cardinal features are initial asymmetrical facial paresthesia and/or sensory deficits followed by bulbar symptoms and spreading of sensory and motor deficits from the face to the scalp, neck, upper trunk, and upper extremities in a rostral–caudal direction. FOSMN is a rare disorder, with only 40–50 reported cases; the exact number of patients is unclear because some reported cases may be overlapping (Barca et al., 2013; Broad & Leigh, 2015; Cruccu et al., 2014; Dalla Bella et al., 2014, 2016; Dobrev et al., 2012; Fluchere et al., 2011; Hokonohara et al., 2008; Isoardo & Troni, 2008; Karakis, Vucic, & Srinivasan, 2014; Knopp, Vaghela, Shanmugam, & Rajabally, 2013; Sonoda et al., 2013; Truini et al., 2015; Vucic et al., 2006, 2012; Ziso et al., 2015). FOSMN is regarded as a neurodegenerative condition with close links to amyotrophic lateral sclerosis (ALS) (Zheng, Chu, Tan, & Zhang, 2016). We and others have reported TAR DNA‐binding protein 43 (TDP‐43)‐positive skein‐like neuronal inclusions together with neuronal loss and gliosis in the spinal anterior horns (Sonoda et al., 2013; Ziso et al., 2015). However, the observations that some patients with FOSMN respond to immunotherapies (Fluchere et al., 2011; Hokonohara et al., 2008; Knopp et al., 2013; Sonoda et al., 2013) and that affected spinal ganglia show numerous Nageotte’s nodules with focal lymphocyte infiltration (Sonoda et al., 2013) suggest possible involvement of an immune‐mediated mechanism. Thus, the precise mechanism of FOSMN remains unclear.

Some patients with FOSMN have a rapidly progressive course (Barca et al., 2013; Broad & Leigh, 2015; Fluchere et al., 2011; Sonoda et al., 2013)_,_ similar to bulbar‐onset ALS (Chiò, Calvo, Moglia, Mazzini, & Mora, 2011). Others have a relatively long course (Cruccu et al., 2014; Dalla Bella et al., 2016; Isoardo & Troni, 2008; Karakis et al., 2014; Vucic et al., 2006, 2012; Ziso et al., 2015), which is unusual for ALS (Haverkamp, Appel, & Appel, 1995). Partly because of the rarity of this disease, long‐term prognostic factors have not been established. Bulbar involvement is frequently observed in this condition (Barca et al., 2013; Broad & Leigh, 2015; Cruccu et al., 2014; Dalla Bella et al., 2014, 2016; Dobrev et al., 2012; Fluchere et al., 2011; Hokonohara et al., 2008; Isoardo & Troni, 2008; Karakis et al., 2014; Knopp et al., 2013; Sonoda et al., 2013; Truini et al., 2015; Vucic et al., 2006, 2012; Ziso et al., 2015) and could be a prognostic factor. However, bulbar manifestations in FOSMN have not been thoroughly described. We previously reported two Japanese FOSMN cases, including the above‐mentioned autopsy case (Hokonohara et al., 2008; Sonoda et al., 2013). After that, four additional patients with FOSMN were thoroughly examined and followed in our clinic. In this study, we aimed to clarify clinical and laboratory features in this series of patients with FOSMN. We used videofluoroscopic swallowing studies (VFSS) and fiberoptic endoscopic evaluation of swallowing (FEES) to evaluate oral phase dysphagia as a critical prognostic factor in FOSMN.

## 2 PATIENTS AND METHODS

### 2.1 Patients and standard protocol approvals

We conducted a retrospective survey of FOSMN cases evaluated in the Department of Neurology at Kyushu University Hospital, Japan, between 1 January 2005 and 30 November 2017. The diagnosis of FOSMN was largely based on the clinical features reported by Vucic et al. (2006). However, there is one report of a patient with FOSMN who suffered from dysarthria and dysphagia at onset followed by sensory symptoms in the face (Broad & Leigh, 2015). Therefore, in this study, the diagnostic and inclusion criteria of FOSMN were as follows: Patients had either (1) slowly progressive sensorimotor symptoms in the face beginning with sensory abnormality or (2) bulbar symptom at onset followed by sensory and motor symptoms in the face, and (3) other disorders were excluded. Patients who fulfilled these inclusion criteria, that is, [(1) or (2)] + (3), were consecutively enrolled in this study as patients with FOSMN. FOSMN was diagnosed in a total of six patients. Clinical information was retrieved by review of medical records. This study was reviewed and approved by the Medical Ethics Committee of Kyushu University.

### 2.2 Evaluation of swallowing

To evaluate the features and severity of dysphagia in our patients with FOSMN, VFSS and/or FEES were performed at the Department of Otorhinolaryngology at Kyushu University Hospital, according to clinical needs. VFSS evaluated the following components: lip closure, tongue control, bolus preparation (oral retention), bolus transport (lingual transfer), oral residue, soft palate elevation, laryngeal elevation, laryngeal closure, pharyngeal stripping wave, pharyngeal esophageal sphincter opening, pharyngeal clearance, esophageal clearance, penetration, and aspiration. FEES findings from videoendoscopy and blue‐dyed water test were scored according to the method of Hyodo et al. (Hyodo’s score), which is widely accepted and used by Japanese otolaryngology specialists because it is useful, simple, and clinically validated (Hyodo, Nishikubo, & Hirose, 2010). In brief, this scoring method was designed to be simple and reliable in daily clinical practice. It consists of the following four parameters: (A) degree of salivary pooling at the vallecular and piriform sinuses, (B) glottal closure reflex induced by touching the epiglottis or arytenoid with the tip of an endoscope, (C) swallowing reflex initiation assessed according to “white‐out” timing, and (D) pharyngeal clearance after swallowing of blue‐dyed water. Each parameter was scored on a scale from 0 to 3, where 0 was normal, 1 was mild impairment, 2 was moderate impairment, and 3 was severe impairment. The total score (0 to 12) was used as an index of swallowing function. In this scoring system, a score greater than 7 indicates a serious risk for aspiration (Hyodo et al., 2010). VFSS and FEES findings were also scored according to the Penetration–Aspiration Scale (PAS), which is a widely used 8‐point clinical scale for rating penetration and aspiration (Colodny, 2002; Rosenbek, Robbins, Roecker, Coyle, & Wood, 1996).

## 3 RESULTS

### 3.1 Case reports

We identified six patients with FOSMN (three women and three men) during the study period. Table 1 summarizes their clinical and demographic features. Average age at onset was 58.5 years (range 44–83 years); average disease duration was 5.7 years (range 2–12 years). All six patients developed sensory impairment in the face, including the oral cavity; sensory impairment spread in a rostral–caudal direction to the scalp, neck, upper trunk, and upper extremities. We briefly describe the clinical course, neurological findings, and results of laboratory tests, imaging, and neurophysiological examinations below.

**Table 1:** Clinical features of six patients with FOSMN

Patient	Age of onset (years)/sex	Disease duration (years)	First symptoms	Sensory disturbance	Motor disturbance	Years from onset to dysphagia	Years from onset to PEG insertion	Immunotherapy and response	Prognosis
1 (Hokonohara et al., 2008)	45/M	12	Dysesthesia in cheek (Rt)	F → scalp → UE	F → neck → UE	6	No	Partial improvement with PE, IVIg, or PSL	Dead (choking)
2 (Sonoda et al., 2013)	45/F	3	Dysesthesia around mouth (Rt)	Rt.F, UE → Trunk	F → UE	1	2	Partial improvement with IVIg or IVMP	Dead (respiratory failure)
3	60/M	9	Numbness around eyes (Bil)	F → scalp → neck, shoulder	Rt.F	2	6	Partial improvement with IVIg	Alive (progressive, repeated aspiration pneumonia)
4	74/F	2	Dysarthria	Lt.F → Rt.F	F → Rt.UE → Rt.LE	0.8	2	Partial improvement with IVIg	Alive (progressive)
5	83/F	3	Dysesthesia in Rt.F	Rt.F → Lt.F	Rt.F	1.8	No	Partial improvement with IVIg	Alive (progressive)
6	44/M	5	Dysarthria	F → UE	F → UE	4	No	No treatment	Alive (progressive)

Note

Bil, bilateral; F, face; IVIg, intravenous immunoglobulin; IVMP, intravenous methylprednisolone; LE, lower extremity; Lt, left; PE, plasma exchange; PEG, percutaneous endoscopic gastrostomy; PSL, prednisolone; Rt, right; UE, upper extremity.

#### 3.1.1 Patients 1 and 2

Patients 1 and 2 were previously reported (Hokonohara et al., 2008; Sonoda et al., 2013). It is worth noting that Patient 1 developed progressive dysphagia and died by choking 12 years after the onset of disease.

#### 3.1.2 Patient 3

##### History

A 60‐year‐old man noticed numbness in his face and oral cavity, which slowly progressed and spread to the scalp, neck, and shoulders. Two years after onset, he developed difficulty swallowing and slurring of his speech. Three years after onset, he was referred to our hospital.

##### Neurological findings

The patient had decreased sensation to pinprick and light touch in all divisions of the trigeminal nerve bilaterally and in the dermatomes of C1–C4. Corneal reflexes were absent. He had weakness and atrophy of the right facial muscles and dysarthria with tongue weakness. Gag reflex was also absent. Chorea‐like involuntary movement was detected in the extremities with right‐sided predominance; however, muscle strength and reflexes of the extremities were normal.

##### Laboratory findings

**Figure 1:**
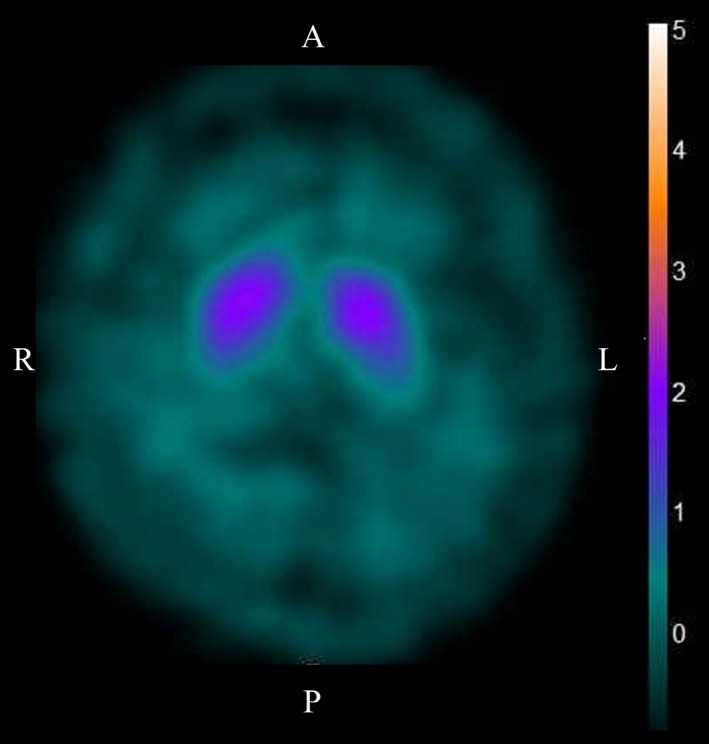
Reduced ^123^I‐ioflupane binding in the bilateral striata of Patient 3. Dopamine transporter SPECT with ^123^I‐ioflupane was performed in Patient 3. ^123^I‐ioflupane binding was reduced in the bilateral striata, with left‐sided predominance. The specific binding ratio was 3.68 on the right and 2.66 on the left side; the asymmetry index was 32.2%

The patient’s blood and cerebrospinal fluid tests were normal. MRI of the brain and spinal cord were also normal. A blink reflex study showed delayed ipsilateral R2 (iR2) and contralateral R2 (cR2) responses bilaterally (latency of iR2 was 50.7 ms, and cR2 was not evoked on left‐sided stimulation; latencies of iR2 and cR2 on right‐sided stimulation were 49.9 and 42.4 ms, respectively; the upper normal limit of R2 latency is <41 ms). A nerve conduction study (NCS) showed decreased sensory nerve action potential amplitudes in the upper limbs. Needle electromyography revealed chronic denervation changes in the sternocleidomastoid muscle. Dopamine transporter SPECT revealed reduced ^123^I‐ioflupane binding in the bilateral striata, with left‐sided predominance (see Figure 1).

##### Diagnosis and treatment

Possible causes of the patient’s chorea‐like movement were ruled out, including Huntington’s disease, chorea‐acanthocytosis, autoimmune diseases, and cerebrovascular diseases. Parkinsonism was not evident. Thus, FOSMN with chorea was diagnosed. Low‐dose haloperidol (1.5 mg/day) relieved the patient’s involuntary movement. The patient was then treated with intravenous immunoglobulin (IVIg) every 3 months, which partially and transiently relieved his sensory symptoms. However, his dysphagia worsened, causing a weight loss of 7 kg in 6 months despite ingestion of minced food and thickened fluids. Percutaneous endoscopic gastrostomy (PEG) insertion was performed for nutritional support 6 years after symptom onset. As PEG insertion, the patient has maintained his weight, although he has had recurrent episodes of aspiration pneumonia.

#### 3.1.3 Patient 4

##### History

A 74‐year‐old woman developed slurred speech; she noticed numbness in her left lip and oral cavity 10 months later. The numbness spread to the right lip over several months. She also noticed difficulty moving her tongue and weakness in her right arm.

##### Neurological findings

Neurological examination 14 months after onset showed decreased sensation to light touch and pinprick in the bilateral trigeminal distributions. The patient also had dysarthria and mild weakness in her face, neck, and right arm, with tongue fasciculations. Light touch, pinprick, vibration, and proprioception of her limbs were all intact. Reflexes, gait, and coordination were normal.

##### Laboratory findings

The patient’s laboratory workup was normal except for the presence of monoclonal protein (IgA‐λ), hepatitis B surface antigen antibody, and hepatitis C virus antibody. A bilateral blink reflex study showed prolonged iR2 and cR2 responses (45.7 and 46.0 ms, respectively, on left‐sided stimulation; 41.8 and 41.3 ms, respectively, on right‐sided stimulation).

##### Diagnosis and treatment

**Figure 2:**
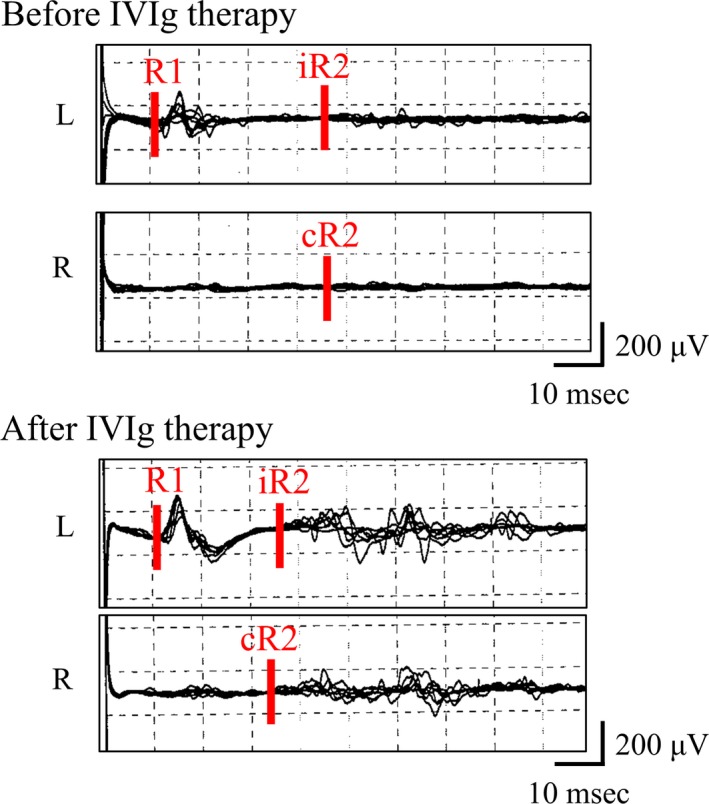
Blink reflex findings before and after immunotherapy in Patient 4. Blink reflex findings with left‐sided stimulation in Patient 4 are shown. Delayed ipsi‐ and contralateral R2 responses (iR2 and cR2, respectively) to left‐sided stimulation improved after intravenous immunoglobulin (IVIg) therapy. The latencies of iR2 and cR2 before treatment were 45.7 and 46.0 ms, respectively; those after treatment were 36.6 and 34.5 ms, respectively

With a diagnosis of FOSMN, the patient received three infusions of IVIg, which relieved her facial numbness completely and improved the delayed iR2 and cR2 responses to left‐sided stimulation in the blink reflex test (36.6 and 34.5 ms, respectively; see Figure 2). However, the patient’s right arm weakness spread to the right leg and her bulbar symptoms worsened. Because she experienced worsening of dysphagia and frequent choking on food, water, and saliva despite modification of food and fluid consistency, resulting in about 5 kg of weight loss over a 6‐month period, a PEG tube was placed 2 years after initial symptoms.

#### 3.1.4 Patient 5

##### History

An 83‐year‐old woman first noticed numbness around the right corner of her mouth, which spread to all areas of the three branches of the bilateral trigeminal nerves. Carbamazepine was prescribed for a presumed diagnosis of trigeminal neuralgia; however, the treatment was not effective. The patient then developed progressive dysarthria and salivation, with difficulty propelling food boluses into the pharynx.

##### Neurological findings

Neurological examination 2 years after onset showed paresthesia in the patient’s whole face and reduced sensation to pinprick and light touch in the left maxillary nerve area. Corneal reflexes were diminished bilaterally. The patient also had dysarthria, mild facial weakness, and tongue weakness. Gag reflex was absent. Muscle strength of the limbs was normal, whereas tendon reflexes were globally brisk with bilateral extensor plantar responses. The patient’s manner of eating was very characteristic. After chewing food, she picked up a mirror in her left hand and checked inside her mouth to identify the food’s location, then moved the food bolus into her pharynx with chopsticks held in her right hand. This manner of eating suggests reduced sensory perception in the oral cavity.

##### Laboratory findings

The patient’s general laboratory workup showed mild hypothyroidism (TSH 7.23 μIU/ml, f‐T4 0.87 ng/dl), with positive antithyroglobulin antibody (193.5 IU/ml) and antithyroid peroxidase antibody (≥600 IU/ml). Other autoantibodies examined were negative, except for positive antinuclear antibody (1:1,280 titer). Blink reflex studies showed prolonged R1 response on the right side (13.6 ms; upper normal limit of R1 latency is <13.0 ms) and prolonged bilateral iR2 and cR2 responses (latencies of iR2 and cR2 were 40.1 and 52.7 ms for left‐sided stimulation and 49.9 and 42.4 ms for right‐sided stimulation, respectively). An NCS was unremarkable.

##### Diagnosis and treatment

The patient was initially treated with levothyroxine, which normalized TSH and f‐T4 levels but did not relieve her symptoms. She was then diagnosed with FOSMN. IVIg therapy was initiated and mildly improved her speech and right‐sided dysesthesia. However, treatment did not improve the patient’s dysphagia.

#### 3.1.5 Patient 6

##### History

A 44‐year‐old man was initially told by others that he could not enunciate well; however, he was not concerned about this change. Two years after onset, he felt numbness around his mouth and had slurring of his speech. Numbness spread to his cheeks, chin, and bilateral fingers within 2 years. Four years after onset, the patient suffered from loss of ability to retain saliva and water within the mouth. He was referred to our hospital 5 years after onset. The patient also had an episode of choking on noodles and being transported to a hospital by ambulance before he underwent detailed examination at our hospital.

##### Neurological findings

Neurological examination showed decreased sensation to pinprick and light touch in all divisions of the trigeminal nerves bilaterally and dysesthesia in the bilateral maxillary and mandibular nerve areas and fingers. Corneal reflexes were absent. The patient had mild weakness and atrophy of the right masseter, facial, and arm muscles. He also had dysarthria, dysphagia, and weakness and atrophy of the tongue with fasciculations. Gag reflex was absent. Reflexes of the extremities were diminished. Gait and coordination were normal.

##### Laboratory findings

The patient’s general laboratory workup was normal. An NCS revealed reduced sensory nerve action potential amplitudes in the bilateral median and ulnar nerves. Needle electromyography showed chronic denervation and reinnervation findings in the trapezius muscles. Left blink reflex studies showed delayed cR2 response (latency 42.9 msec), with normal responses to right‐sided stimulation.

##### Diagnosis and treatment

Based on his clinical history and findings, the patient was diagnosed with FOSMN. He was followed up but chose to receive no treatment.

### 3.2 Dysphagia and evaluation of swallowing

All patients experienced bulbar symptoms with dysphagia early in the course of the disease (bulbar involvement at an average of 1.8 years [range, 0 to 6 years] from onset; dysphagia at an average of 2.6 years [range, 10 months to 6 years] from onset). Four patients (Patients 2–5) developed dysphagia within 2 years; bulbar involvement preceded sensory disturbance in Patients 4 and 6. Because of dysphagia, three patients had choking episodes (Patients 1, 4, and 6, causing death in Patient 1), three patients needed PEG insertion (Patients 2, 3, and 4), and one had recurrent episodes of aspiration pneumonia (Patient 3). At the time of the swallowing function tests, tongue weakness was severe in Patient 4; moderate in Patients 2 and 6; and mild in Patients 1, 3, and 5.

Table 2 summarizes the VFSS and FEES findings. VFSS was performed in all patients except Patient 6. The test uncovered poor oral retention in Patients 1, 2, 4, and 5 and poor lingual transfer in Patient 3. Poor oral retention resulted in boluses flowing into the pharynx before swallowing in Patients 2 and 4. Moreover, Patients 2 and 3 showed mild aspiration after or during swallowing, resulted in relatively high PAS scores (6 in both) on VFSS. In Patients 3 and 4, Hyodo’s scores were low despite the clinical need for PEG insertion. Although Patient 5 also had difficulty eating food, as described above, her Hyodo’s score was 2, suggesting that the pharyngeal phase of swallowing was only minimally impaired. Despite a Hyodo’s score of 2 in Patient 6, he had an episode of choking. Of the five patients examined with FEES, leakage of blue‐dyed water from the mouth to the pharynx because of poor oral retention was observed in three (Patients 2, 3, and 6); this leakage was not counted in Hyodo’s scoring (Hyodo et al., 2010). In PAS scoring of FEES, neither penetration nor aspiration was detected in any patient (Patients 3–6). These findings suggest that patients with FOSMN had dysphagia that was more predominant in the oral phase than the pharyngeal phase, especially in the early course of the disease. The findings also indicate that the risk of aspiration in patients with FOSMN can be underestimated if only the easy‐to‐use scoring systems of FEES are used.

**Table 2:** Evaluation of swallowing in six patients with FOSMN

Patient	Timing (years from onset)	Videofluoroscopic swallowing study				Fiberoptic endoscopic evaluation of swallowing			
		PAS score	Oral retention	Lingual transfer	Note	Hyodo’s score^a^ (A/B/C/D)	PAS score	Leakage of blue‐dyed water	Note
1^b^	L (10)	1	Poor	NM	Poor pharyngeal clearance	NE	NE	NE	NE
2^b^	P (2.5)	6	Poor	NM	Material flow into pharynx prematurely; aspiration after swallowing	NA (abnormal in part A, C and D)	NA	Yes	Nasal regurgitation
3	P (6)	6	Normal	Slightly poor	Mild aspiration during swallowing	4 (1/0/2/1)	1	Yes	
4	P (2)	2	Poor	Normal	Material flow into pharynx prematurely	4 (1/0/1/2)	1	No	
5	L (2)	1	Poor	Normal	Normal in pharyngeal phase	2 (0/0/2/0)	1	No	
6	L (5)	NE	NE	NE	NE	2 (1/1/0/0)	1	Yes	

Note

L, latest evaluation; NA, not applicable; NE, not examined; NM, not mentioned; P, evaluated just before insertion of percutaneous endoscopic gastrostomy; PAS, Penetration–Aspiration Scale.

^a^Total scores and subscores (in brackets) are based on Hyodo’s score (Hyodo et al., 2010), described in the Section 2. ^b^Patients 1 and 2 were evaluated before the use of Hyodo’s endoscopic scoring system.

## 4 DISCUSSION

The main new findings of the present study on FOSMN are as follows. (1) Early bulbar manifestations, presenting as the cardinal symptoms of dysphagia, choking, and aspiration pneumonia, are common in this condition. (2) The main mechanism of dysphagia is oral phase and not pharyngeal phase dysphagia, necessitating early PEG insertion despite low FEES scores. (3) Although IVIg can partially and transiently alleviate sensory impairment, relentless progression of motor weakness, culminating in death, is unaffected by immunotherapy. (4) Patients with FOSMN occasionally present with involuntary movement.

Consistent with a previous report (Vucic et al., 2006), all our patients developed sensory impairment in the face, spreading in a rostral–caudal direction. Bulbar involvement preceded sensory disturbance in Patients 4 and 6, which is consistent with the report by Broad and Leigh (Broad & Leigh, 2015). We thus consider all our cases to fit the category of FOSMN. All patients developed bulbar dysfunction with dysphagia early in the course of the disease, which resulted in choking, PEG insertion, recurrent aspiration pneumonia, and even death. Therefore, we consider bulbar involvement one of the most life‐threatening factors in FOSMN, along with respiratory failure, as seen in Patient 2. According to a survey of the literature that does not include the present cases, nine patients died an average of 3.4 years after symptom onset; the identified causes of death were aspiration pneumonia and/or respiratory insufficiency (Barca et al., 2013; Broad & Leigh, 2015; Fluchere et al., 2011; Truini et al., 2015; Vucic et al., 2006; Ziso et al., 2015).

We judged bulbar involvement in our patients to be severe, according to their complaints and clinical signs such as weight loss, prolongation of mealtime, and decreased food consumption. However, the swallowing findings according to Hyodo’s FEES scoring in our patients were unexpectedly mild. This discrepancy can be explained by the fact that Hyodo’s score focuses on the pharyngeal phase of swallowing, whereas patients with FOSMN predominantly have difficulty in the oral phase. Hyodo’s total scores are useful for evaluating the ability of oral food intake: subjects with total scores below 4 or 5 have satisfactory oral feeding ability, whereas those with total scores above 9 or 10 have difficulty with oral feeding (Hyodo et al., 2010). Despite these previous findings, the Hyodo’s scores of patients with FOSMN who needed PEG insertion or had episodes of choking were between 2 and 4, which should have been adequate for oral feeding. We did not use the Langmore score (Langmore, 2001) to evaluate oral phase dysphagia in the present study because the enormous quantity of data it requires makes it impractical in clinical practice. Instead, we evaluated leakage of blue‐dyed water from the mouth to the pharynx with FEES as well as oral retention and lingual transfer with VFSS. VFSS disclosed four cases of poor oral retention and premature material flow into the pharynx, which can cause aspiration. Leakage of blue‐dyed water, indicating poor oral retention, was also observed with FEES in three cases. Thus, all of the present patients with FOSMN were confirmed to have deficits in oral retention by either FEES or VFSS. At the time of the swallowing function tests, tongue weakness was mild to moderate in all but one patient in our FOSMN series. Thus, poor oral retention may well have arisen from sensory impairment and numbness in the oral cavity in addition to weakness of the tongue, masseter, and perioral muscles.

The pathological findings of our autopsy case (Patient 2) showed loss of neurons and reactive gliosis with TDP‐43‐positive glial inclusions not only in the motor nuclei of the trigeminal and hypoglossal nerves but also in the trigeminal main sensory nucleus and spinal trigeminal tract (Sonoda et al., 2013). Similar pathological findings with neuronal loss and reactive gliosis have been reported in the motor and sensory nuclei of the trigeminal nerve, facial nerve nucleus, dorsal motor nucleus of vagal nerve, ambiguous nucleus, and hypoglossal nucleus (Vucic et al., 2006, 2012; Ziso et al., 2015). These pathological changes of the pontine and medullary cranial nerve nuclei may well explain impairment of oral retention and dysphagia in patients with FOSMN. Therefore, poor oral retention and premature material flow resulting in oral phase dysphagia appear to be characteristic findings in FOSMN.

By contrast, patients with ALS usually develop not only oral but also pharyngeal phase dysphagia when they require modification of foods or tube feeding (Higo, Tayama, & Nito, 2004). Patients with spinal and bulbar muscular atrophy (SBMA), another type of motor neuron disease, also develop dysphagia in both oral and pharyngeal phases. In patients with SBMA, pharyngeal phase abnormalities are more frequently observed than oral phase abnormalities (Banno et al., 2017). Patients with Parkinson’s disease, one of the most common neurodegenerative diseases, also frequently develop dysphagia. The most characteristic finding of dysphagia in Parkinson’s disease is residues after swallowing; leakage from the oral cavity before swallowing is not frequently observed in these patients (Pflug et al., 2018). Therefore, the findings of dysphagia in patients with FOSMN seem to be relatively unique to this condition. According to our literature survey, bulbar involvement was present in at least 43 of 47 FOSMN cases (91%) (Barca et al., 2013; Broad & Leigh, 2015; Cruccu et al., 2014; Dalla Bella et al., 2014, 2016; Dobrev et al., 2012; Fluchere et al., 2011; Hokonohara et al., 2008; Isoardo & Troni, 2008; Karakis et al., 2014; Knopp et al., 2013; Sonoda et al., 2013; Truini et al., 2015; Vucic et al., 2006, 2012; Ziso et al., 2015). Based on the present findings, we propose that bulbar involvement manifesting with dysphagia could be a critical prognostic factor in FOSMN and that oral phase dysphagia should be carefully evaluated in patients with FOSMN.

We found discrepancy in PAS scores between VFSS and FEES in Patient 3. This discrepancy could have been caused by methodological differences. In VFSS, movement of the swallowed material is visualized and recorded during the entire process of swallowing, including the preswallowing period, and can be retrospectively reviewed. In FEES, passage of the bolus cannot be seen during swallowing because of “white‐out”; only the areas visualized by videoendoscopy are recorded and evaluated. As a result, if aspiration before swallowing does not catch the observer’s attention, it cannot be retrospectively reviewed with FEES. Therefore, when FEES is used to test swallowing function in patients with FOSMN, premature material flow from the oral cavity to the pharynx should be carefully assessed.

All our patients partially and transiently responded to IVIg; however, progression of motor weakness was relentless. Some patients with FOSMN, including our cases, harbor autoantibodies (Dobrev et al., 2012; Knopp et al., 2013; Sonoda et al., 2013; Vucic et al., 2006, 2012) and partially respond to various immunotherapies (Fluchere et al., 2011; Hokonohara et al., 2008; Knopp et al., 2013; Sonoda et al., 2013). Together with the pathological observation of lymphocytic infiltration in peripheral nerves, cervical root ganglion, and ventral nerve root in one autopsy case (Sonoda et al., 2013), these findings suggest an inflammatory component to this condition. However, neuronal loss and gliosis in the nuclei of affected cranial nerves, including the trigeminal nucleus and tract, dorsal root ganglia, anterior horn cells, and anterior spinal roots with or without TDP‐43‐positive inclusions (Sonoda et al., 2013; Vucic et al., 2006, 2012; Ziso et al., 2015), indicate the essential neurodegenerative nature of this disease. Therefore, it should be emphasized that early intervention for dysphagia is important in managing patients with FOSMN.

One of our patients had chorea‐like involuntary movement from the onset. Although this involuntary movement could have been incidental, a patient from another institute in Japan was also reported to have chorea‐like hyperkinesis (Taguchi et al., 2016). In our patient, haloperidol effectively controlled involuntary movements and ^123^I‐ioflupane binding was decreased in the striata, suggesting that presynaptic dopaminergic abnormality was the underlying mechanism of this chorea (Booth et al., 2015a,b). It is possible that chorea is a rare manifestation of FOSMN, in which multiple systems may undergo degeneration. Careful observation of larger numbers of patients with FOSMN is required to clarify this issue.

FOSMN is a developing disease without clearly established diagnostic criteria. We chose the inclusion criteria in this study on the basis of previously reported clinical features of patients with FOSMN (Barca et al., 2013; Broad & Leigh, 2015; Cruccu et al., 2014; Dalla Bella et al., 2014, 2016; Dobrev et al., 2012; Fluchere et al., 2011; Hokonohara et al., 2008; Isoardo & Troni, 2008; Karakis et al., 2014; Knopp et al., 2013; Sonoda et al., 2013; Truini et al., 2015; Vucic et al., 2006, 2012; Ziso et al., 2015). Moreover, we consecutively enrolled all patients who fulfilled the criteria in the present study. Therefore, we believe that there was no serious selection bias that could have distorted our conclusions. However, selection bias cannot be completely ruled out because this study was retrospective and patient enrollment was based on our own inclusion criteria.

In conclusion, bulbar involvement with oral phase dysphagia can be a life‐threatening prognostic factor in patients with FOSMN; this symptom is manageable with early PEG intervention. Neurologists should be aware of this condition and pay particular attention to dysphagia.

## CONFLICT OF INTEREST

MW has received a grant from GlaxoSmithKline. RY has received research support from Bayer Schering Pharma, Biogen Idec Japan, Novartis Pharma, and Mitsubishi Tanabe Pharma. NI has received a grant and salary from Mitsubishi Tanabe Pharma, Bayer Yakuhin, and Japan Blood Products Organization. JK is a consultant for Biogen Idec Japan, Novartis Pharma AG, Medical Review, Chugai Pharmaceutical, and Teijin Pharma. He has received speaking fees and/or honoraria from Bayer Healthcare, Mitsubishi Tanabe Pharma, Nobelpharma, Sanofi, Otsuka Pharmaceutical, Novartis Pharma KK, Takeda Pharmaceutical Company, Nippon Rinsho, Pfizer, Eisai, and Medical Review. The other authors have nothing to disclose and no conflict of interests.
